# Patient-Specific Mathematical Neuro-Oncology: Using a Simple Proliferation and Invasion Tumor Model to Inform Clinical Practice

**DOI:** 10.1007/s11538-015-0067-7

**Published:** 2015-03-21

**Authors:** Pamela R. Jackson, Joseph Juliano, Andrea Hawkins-Daarud, Russell C. Rockne, Kristin R. Swanson

**Affiliations:** 1 Mathematical NeuroOncology Lab, Department of Neurological Surgery, Northwestern University Feinberg School of Medicine, 676 N St Clair Street, Suite 1300, Chicago, IL 60611 USA; 2 Department of Engineering Sciences and Applied Mathematics, Northwestern University, Evanston, IL USA

**Keywords:** Glioblastoma, Mathematical model, Patient-specific

## Abstract

Glioblastoma multiforme (GBM) is the most common malignant primary brain tumor associated with a poor median survival of 15–18 months, yet there is wide heterogeneity across and within patients. This heterogeneity has been the source of significant clinical challenges facing patients with GBM and has hampered the drive toward more precision or personalized medicine approaches to treating these challenging tumors. Over the last two decades, the field of Mathematical Neuro-oncology has grown out of desire to use (often patient-specific) mathematical modeling to better treat GBMs. Here, we will focus on a series of clinically relevant results using patient-specific mathematical modeling. The core model at the center of these results incorporates two hallmark features of GBM, proliferation $(\rho )$ and invasion (*D*), as key parameters. Based on routinely obtained magnetic resonance images, each patient’s tumor can be characterized using these two parameters. The Proliferation-Invasion (PI) model uses $\rho$ and *D* to create patient-specific growth predictions. The PI model, its predictions, and parameters have been used in a number of ways to derive biological insight. Beyond predicting growth, the PI model has been utilized to identify patients who benefit from different surgery strategies, to prognosticate response to radiation therapy, to develop a treatment response metric, and to connect clinical imaging features and genetic information. Demonstration of the PI model’s clinical relevance supports the growing role for it and other mathematical models in routine clinical practice.

## Introduction

Mathematical modeling has provided insight into a vast array of complex systems. The applications to weather, climate, and financial markets represent a small fraction of the many areas that rely extensively on mathematics. From generating hypotheses, to prediction of results later validated by experiment, mathematics provides a framework that has elucidated non-intuitive and nonlinear phenomenon.

In medicine, practitioners regularly attempt to manage disease that manifests nonlinearly. Because of this, medicine, in the most general sense, is considered both an art and science. At the interface, medical art concerns the clinical practice by which a physician manages the unpredictability of individual patients while medical science concerns general trends in observed population outcomes. This was well understood as far back as 1892 where Canadian physician Sir William Osler stated “If it were not for the great variability among individuals, medicine might as well be a science, not an art.”

However, today there exists a barrage of genomic, epigenetic, and imaging data that can be generated for each individual patient, allowing for the potential to determine how individuals may respond to treatment that can be personalized to their individual variability. As medical science seeks to study individual variability to inform treatment practice, mathematics is being employed in order to elucidate how disease may progress, how individual variability can affect treatment, and how to design better treatment regimes.

Among many areas in medicine, mathematics has provided particular insights into the most common form of primary brain tumors, glioblastoma multiforme (GBM). GBM is a very aggressive form of cancer with uniformly poor patient prognosis (Louis et al. 2007; Ostrom et al. 2013). Over the past decade, standard treatment regimens have modestly increased median survival to 15–18 months (Stupp 2007). However, population-level data are poor predictors of individual patient outcome due to the heterogeneity of patient response to treatment (Johnson and O’Neill 2012). Currently, prognosis, and response to treatment for individual patients are largely based upon histopathologically similar subsets of patients. These subsets are currently unable to characterize the full extent by which patient heterogeneity may influence his or her individual treatment response. This paper aims to highlight how patient-specific mathematical modeling is helping to move neuro-oncology toward developing individualized treatment rather than measuring patient success against the statistically average patient. Specifically, we will show how models of GBM can evaluate treatment efficacy, predict survival outcomes, and examine links between clinical imaging and genetics that can be used to provide clinical tools for optimally treating each individual patient.

## Building a Minimal Model with Sufficient Complexity to Predict Patient-Specific Tumor Growth

Advances in DNA sequencing and genomics have led to a greater understanding of cancer complexity. Hanahan and Weinberg (2000) described cancer cells as generally having a set of six hallmarks: limitless replicative potential, sustained angiogenesis, evading apoptosis, self-sufficiency in growth signals, insensitivity to anti-growth signals, and tissue invasion and metastasis (Hanahan and Weinberg 2000). While much of the research regarding cancer details the myriad of mechanisms that have been deregulated or otherwise gone wrong, it is clear that the majority of the hallmarks of cancer can be distilled down to say that cancer is defined by “cells that proliferate abnormally and migrate to distant regions.” In particular, GBMs are notorious for exhibiting these two phenomena as they both aggressively proliferate and diffusively invade the brain, although interestingly they do not metastasize to other organs generally. Over the last decade, the Swanson lab has utilized a mathematical model, referred to as the Proliferation-Invasion (PI) model, which captures these two key elements. This minimal model is manifested mathematically as:

$$\begin{aligned} \overbrace{\frac{\partial c}{\partial t}}^{\begin{array}{c} \hbox {Rate of}\\ \hbox {change of}\\ \hbox {glioma cell density}\\ \end{array}}= \overbrace{\nabla \cdot (D\nabla c)}^{\begin{array}{c} \hbox {Net dispersal}\\ \hbox {of giloma cells}\\ \end{array}}+\overbrace{\rho c\left( {1-\frac{c}{K}} \right) }^{\begin{array}{c} \hbox {Net prolif eration}\\ \hbox {of giloma cells}\\ \end{array}} \end{aligned}$$

where c is concentration of tumor cells $(\hbox {cells/mm}^{3})$, *D*(*x*) is the net rate of diffusion ($\hbox {mm}^{2}$/year), and $\rho$ is the net rate of proliferation (1/year) (Swanson et al. 2002; Swanson 1999). The PI model can be used to simulate future growth of the tumor if left untreated based on growth kinetics measured from clinically available magnetic resonance images (MRIs). The simulated future growth can be considered as an untreated virtual control (UVC) when compared to a patient’s treated tumor. In general, the use of this model implies that there are tumor cells everywhere throughout the brain, just in different concentrations, and that there is an underlying traveling wave phenomena resulting in the tumor radius growing linearly over time. The real power of this model, however, comes in the ability to personalize the model parameters to each patient, i.e., in the individualization of the net rates of proliferation $(\rho )$ and invasion (*D*). Through a few key assumptions, regarding spherical symmetry and cellular density thresholds corresponding to different MRI sequences, the Swanson group has determined a technique for estimating these patient-specific parameters (Swanson et al. 2010, 2008). By using these calibration techniques, the PI model has been able to provide predictions of outcomes following surgical resections (Swanson et al. 2003; Baldock et al. 2014a), chemotherapy (Swanson et al. 2002, 2003), radiation (Rockne et al. 2010), and IDH1 mutation status (Baldock et al. 2014b). Here, we highlight some successes of this modeling approach.

## Patient-Specific Mathematical Models Identify Patients Who Will Derive the Greatest (and Least) Benefit from Surgery

Surgery is typically the first intervention given to GBM patients. As GBM is intrinsically a diffuse disease, the concept of surgical resection as a primary treatment modality for this disease has been long debated as it is unclear how much of the total tumor resection can be achieved. Over the last decade, a series of large-scale studies of several hundred patients each have shown that a few months increase in the median survival is achieved if most of the imageable tumor is resected before any additional therapies (Lacroix et al. 2001; McGirt et al. 2009; Sanai et al. 2011; Stummer et al. 2011; Zinn et al. 2013). Current definitions of gross total resection (GTR) and subtotal resection (STR) are dependent on imaging (Shinoda et al. 2001); however, the invasive nature of glioma results in boundaries that are difficult to define not only on imaging but also in the operating room. The relative success of any tumor resection is dependent on a how much of the difficult to define tumor invasion has been removed.

Swanson et al. (2008) utilized the PI model to predict survival following a resection (Swanson et al. 2008). Initial tumor size, treatment response, and subsequent regrowth were modeled for patients receiving either a gross total resection, a subtotal resection or a biopsy (Swanson et al. 2008). After assuming a uniform fatal size tumor, Swanson et al. (2008) were able to accurately predict the survival curves for virtual patients with a range of sizes and extent of resections. No significant difference was found between the survival curves for actual and simulated patient tumor growth (*p* = 0.7, $\chi ^{2}$-test) (Swanson et al. 2008).

**Fig. 1:**
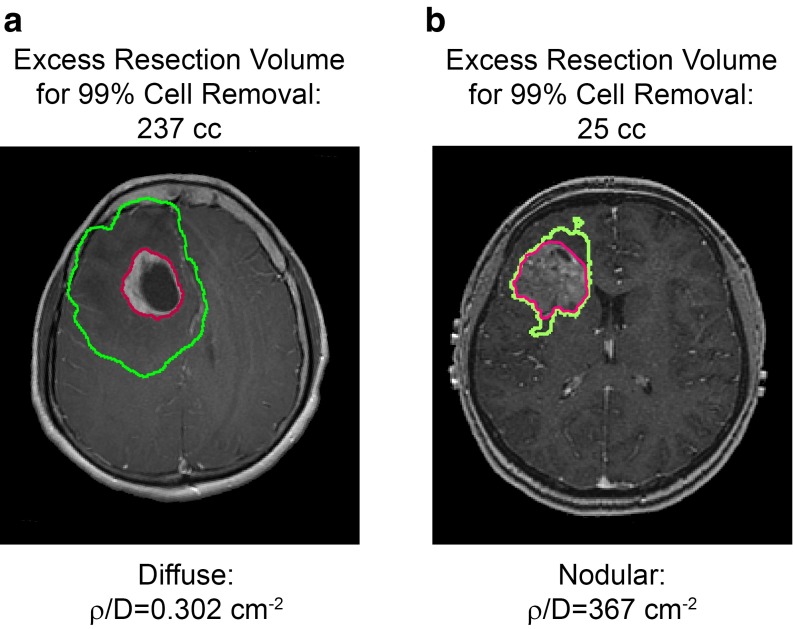
**a** Example “diffuse” tumor annotated with GTR margin (*red*) and model predicted margin (*green*) needed to remove 99 % of tumor cells and **b** example “nodular” tumor annotated with GTR margin (*red*) and model predicted margin (*green*) needed to remove 99 % of tumor cells. The closer the GTR and model predicted 99 % margins are, the better the survival outcomes for patients receiving a GTR. Adapted from (Baldock et al. 2014a)

In a study of 243 newly diagnosed GBMs, Baldock et al. (2014a) further examined how the patient-specific model parameters ($\rho$ and *D*) were related to the patient’s survival following resection (Baldock et al. 2014a). Specifically, the hypothesis tested was that GTR of the “tip-of-the-iceberg” of a more diffuse tumor (low $\rho$/*D*) will leave more residual tumor cells behind than GTR of a less diffuse, more nodular tumor (high $\rho$/*D*). Patients were grouped according to relative invasiveness which was quantified as $\rho$/*D*, where “low” $\rho$/*D* indicates diffuse disease that was more invasive than proliferative, “moderate” indicating that the disease was similarly invasive and proliferative, and “high” $\rho$/*D* indicates nodular disease that was more proliferative than invasive. Out of the population of patients with “high” $\rho$/*D* or nodular tumors, those receiving a GTR had a nearly 8-month improvement in median survival over those who received a STR or biopsy (*p* = 0.0014, log-rank test; Baldock et al. 2014a). However, there was no significant survival difference between GTR and STR/biopsy for patients with low (*p* = 0.53, log-rank test) or moderate (*p* = 0.45, log-rank test) $\rho$/*D* tumors (Baldock et al. 2014a). Thus, patients with high *p*/*D* (nodular tumors) had a significant difference in survival after GTR compared with diffuse or moderate *p*/*D* tumors (Fig. 1). Understanding which patients will benefit most from surgery may affect how and when concomitant therapies are given.

## Patient-Specific Mathematical Models Predict Response to Radiation Therapy in Individual Patients

After resection, radiation therapy is considered part of the standard of care for treating gliomas. Predicting individual differences in radiation response across patients remains elusive, and, as such, all patients of a similar grade receive a similarly designed radiation treatment of roughly 60 Gy delivered over 6 weeks of therapy. In recent years, mathematical modeling has been applied to estimate treatment response in a patient-specific manner. Specifically, Rockne et al. (2009) introduced the medically applicable linear quadratic model into the PI model in order to examine the effectiveness of the standard radiation protocol compared with alternative dosing schedules (Rockne et al. 2009). Results indicated that the optimal response was a low-frequency, high-dose schedule similar to gamma knife procedures used today.

**Fig. 2:**
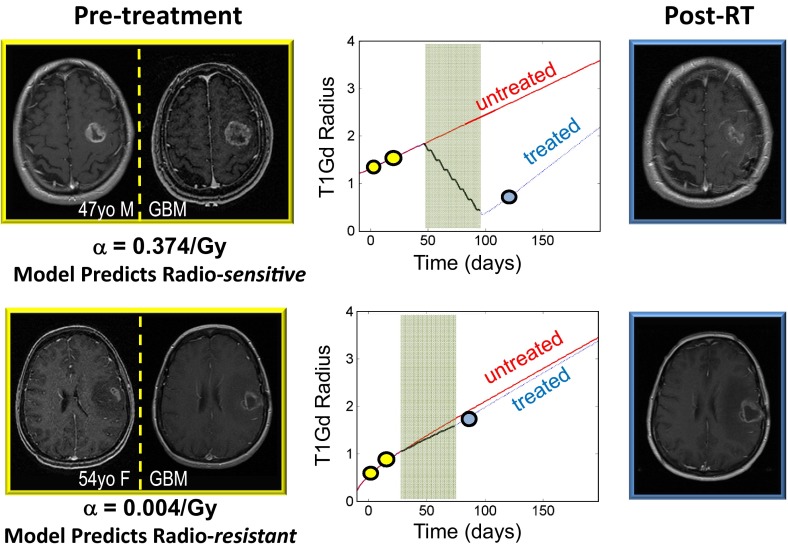
Pre-treatment MRI’s (*yellow boxes*) used to calculate alpha parameter from the linear-quadratic model. *Top* patient has an alpha parameter of 0.340 /Gy which the model predicts is radio-sensitive. *Bottom* patient (0.016 /Gy) is predicted to be radio-resistant. Tumor growth curves show top patient post-treatment MRI tumor radius (*blue dot on blue line*) is highly deflected from the UVC simulation (*red line*). Bottom patient post-treatment MRI tumor radius is hardly deflected from the UVC. Post-treatment MRI’s (*blue boxes*) show that the model-predicted radio-sensitive patient’s tumor is much more responsive to treatment than the model-predicted radio-resistant patient

Taking the model one step further, Rockne et al. (2010) applied the Proliferation-Invasion-Radiation-Therapy (PIRT) model on nine individual patients and found that radiation response in patients may be predicted prior to treatment (Fig. 2; Rockne et al. 2010). Specifically, using a leave-one-out cross-validation technique, the authors found that the net proliferation parameter $(\rho )$ determined from clinical imaging was highly correlated with the radiation response parameter in the linear quadratic model (*r* = .89, *p* = 0.0007). The feasibility of the model gives physicians the ability to take already-used metrics in the clinic and apply them into the PIRT model in order to help determine how well a patient may respond to radiation therapy.

In this case, mathematics made a contribution to radiation biology. The patient-specific PIRT model quantified and elucidated the relationship between the net proliferation rate $(\rho )$ of cancer cells and the sensitivity of the tumor to radiation therapy. This is due to the increased susceptibility of cells to radiation-induced DNA damage during cell mitosis, which is positively correlated with the net proliferation rate $(\rho )$ of the lesion. Although this relationship has been previously studied in cell culture and in animal models, the patient-specific PIRT mathematical model enabled the data-driven validation of this radiation biology truism in human data. Further, these data provide the basis for ongoing investigations in the form of clinical trials that may ultimately be used to migrate away from one-size-fits-all sub-optimal protocols and toward patient-specific optimized radiation therapy strategies that may maximally benefit each patient.

## Patient-Specific Mathematical Models Predictably Connect Patient-Specific Metrics of Treatment Response with Patient Survival

**Fig. 3:**
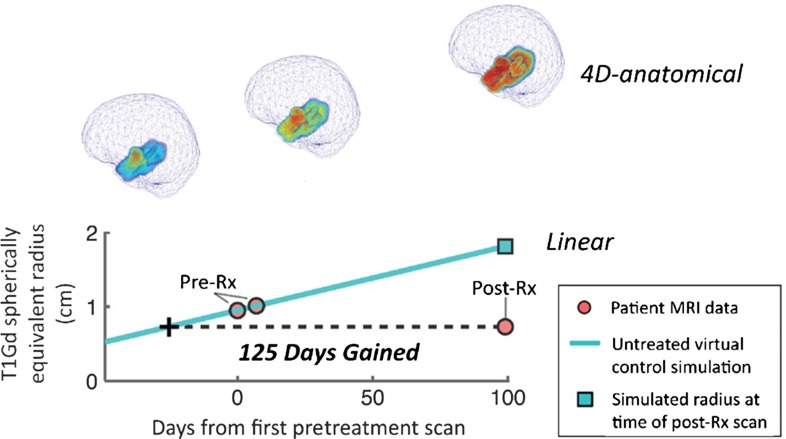
Calculation of the Days Gained score. *Blue line* is the UVC simulated based on the pretreatment patient MRI data (*pink dots on blue line*). The one-dimensional straight-line distance from UVC to the post-treatment MRI point is the Days Gained score. The four-dimensional anatomical brain tumor is shown in its native position within the patient’s brain. *Color* indicates density of tumor cells where *red* is high density. Adapted from (Neal et al. 2013a)

Glioblastomas are known to be particularly resistant to therapy. This is compounded by the fact that current clinical response metrics fail to connect measures of treatment response with patient outcomes (i.e., overall survival). By incorporating a patient-specific model prediction of the tumor growth course if the tumor had gone untreated, Neal et al. (2013a, b) devised a metric known as Days Gained to estimate the effectiveness of therapy for individual patients (Neal et al. 2013a, b). The Days Gained metric is defined through a comparison of the post-treatment lesion size with the UVC growth profile, and indicates the number of days of growth that have been deflected due to therapy. Patients with high Days Gained scores indicate that their tumor growth rate was highly reduced after therapy, while low Days Gained scores indicate their tumor growth rate changed very little in response to therapy (Fig. 3). We emphasize that Days Gained does not actually indicate the amount of time a patient will live longer due to therapy, but instead, serves as metric to determine how effective a given treatment deflected a tumor off its untreated growth curve (Fig. 3).

Another clinical application of Days Gained is that it may help physicians distinguish tumor progression from pseudoprogression, which affects 20–30 % of patients and is defined as an increase in apparent tumor mass that spontaneously subsides without treatment (Pope and Hessel 2011). This is a particularly difficult problem in the clinical setting because the changes seen on imaging may not actually represent the patient’s underlying response to future treatment. Thus, treatment may be stopped or changed based on pseudoprogression under the mistaken assumption that the patient did not respond to treatment. The Days Gained metric was recently applied to 63 patients, and under certain conditions was found to distinguish pseudoprogression from true progression (Neal et al. 2013a). Unlike existing clinical response criteria such as Response Assessment in NeuroOncology—RANO, MacDonald, and Response Evaluation Criteria in Solid Tumors—RECIST, Days Gained utilizes patient-specific tumor kinetics in order to quantify treatment response. Perhaps one of the best advantages is that the metric requires no new available clinical imaging than that routinely done for each patient and can be incorporated into the existing clinical routine**.**

## Patient-Specific Mathematical Models Elucidate Connection Between Key Mutations and Tumor Cell Kinetic Rates

The presence of an IDH1 mutation has been associated with favorable prognosis for GBM patients (Hartmann et al. 2010). Patients with the mutated version of the gene have an average survival of 3.8 years which was significantly longer survival time than patients with the wild-type version (1.1 years; Nobusawa et al. 2009; Parsons et al. 2008). Pre-surgical/pre-treatment indication of IDH1 status could be important for the management of GBMs; however, IDH1 status cannot be determined until after surgery or biopsy.

The PI model parameters $\rho$ and *D*, which are parameterizations of the tumors proliferation and diffusion, are related to tumor aggressiveness. The two parameters can be combined to form an aggressiveness ratio $\rho$/*D*, where high $\rho$/*D* is more indicative of fast-growing GBM with little invasion, and low $\rho$/*D* is representative of a slower-growing GBM with a high degree of invasion. This aggressiveness ratio has previously been correlated with poor prognosis (Wang et al. 2009) and increased hypoxia (Szeto et al. 2009), both related to tumor aggressiveness. Based on these insights, IDH1 mutant tumors were expected to have lower $\rho$/*D* than IDH1 wild-type tumors.

**Fig. 4:**
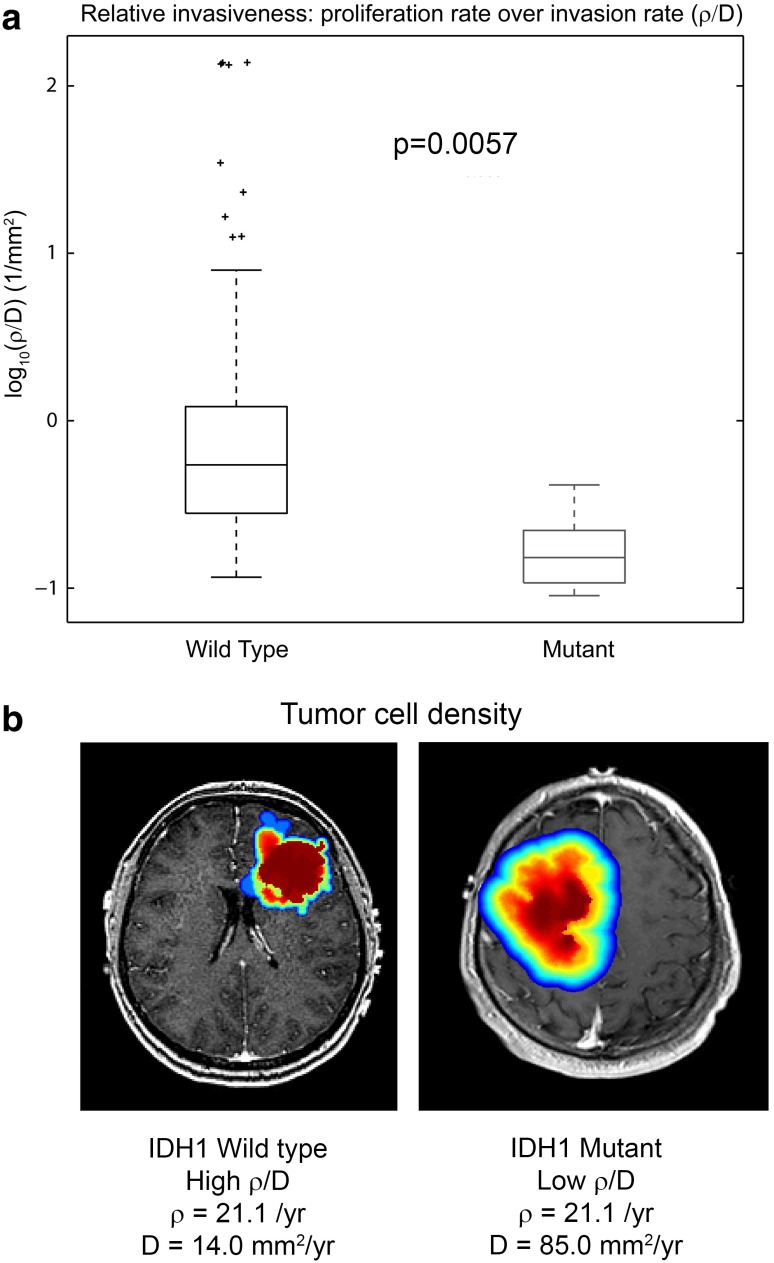
**a** The range of $\rho$/*D* values for IDH1 wild-type and mutant tumors. The mean value of $\rho$/*D* is significantly different between wild-type (*n* = 42) and mutant (*n* = 11) tumors (*p* = 0.0057, *t* test). **b** Example IDH1 wild-type and mutant tumors with model-predicted tumor cell density overlay where *blue* is low tumor cell density and *red* is high tumor cell density. Adapted from (Baldock et al. 2014b)

Baldock et al. (2014b) found that $\rho$/*D* can be used to predict IDH1 status. As shown in Fig. 4, IDH1 mutant tumors had a significantly lower mean $\rho$/*D* than non-mutant tumors (*p* = 0.0057, *t* test) (Baldock et al. 2014b). Using a threshold for $\rho$/*D*, IDH1 mutation status could be predicted with 81.3 % sensitivity and 85.9 % specificity. Conversely, IDH1 wild-type tumors were also accurately predicted with 85.9 % sensitivity and 81.3 % specificity. Compared to IDH1 wild-type tumors, IDH1 mutant tumors had a relatively more diffuse growth pattern, which was likely related to their low $\rho$ and high *D* values (Baldock et al. 2014b). Both $\rho$ and *D* were compared individually between the two groups, and $\rho$ was found to be significantly lower in IDH1 mutant tumors (*p* = 0.046, *t* test), but no difference was found for *D* (*p* = 0.503, *t* test; Baldock et al. 2014b). Likely, the growth pattern of mutant tumors was more driven by the lowered proliferation rather than increased invasion as compared to IDH1 wild-type tumors. The predictive capability of $\rho$/*D* for IDH1 status could be utilized for clinical decisions pre-operatively.

## Looking Forward: What Will Math Do Next for Neuro-Oncology?

The PI model has been used to derive biological insight in order to predict growth, evaluate treatment efficacy, delineate response to treatment, and examine links between clinical imaging and genetic information. This has been made possible because the mathematical modeling framework allows exploration of a minimal set of biological mechanisms at play and connects them to a higher level understanding. While the examples provided here have been focused on patient-specific utilizations, models can be used in broader ways. As an example, we have made efforts to expand the PI model to include information regarding hypoxia, necrosis, and angiogenesis (Swanson et al. 2011). This model is not yet capable of patient-specific prediction; however, we have used this model to consider disease-level questions regarding patterns of malignant progression (Swanson et al. 2011) and response to anti-angiogenic therapy as seen on imaging (Hawkins-Daarud et al. 2013).

As medicine strives to become increasingly personalized, such successes, as presented here, support a growing role for mathematical modeling in the clinic. The Days Gained metric described here has already been tested in the context of clinical trials for predicting outcomes in terms of overall survival (Adair et al. 2014). In addition, efforts are underway to investigate how mathematical models can be used to create virtual clinical trials for enhancing results from trials with a low number of patients. From identifying IDH1 mutations to predicting radiation response, mathematical modeling has already demonstrated a potential for clinical effectiveness. This, however, is just the tip of the iceberg; mathematical models have and will continue to enable more discoveries in biology and medicine, and vice versa.
